# Tumor-Targeted Immunotherapy by Using Primary Adipose-Derived Stem Cells and an Antigen-Specific Protein Vaccine

**DOI:** 10.3390/cancers10110446

**Published:** 2018-11-15

**Authors:** Jui-Hua Lu, Bou-Yue Peng, Chun-Chao Chang, Navneet Kumar Dubey, Wen-Cheng Lo, Hsin-Chung Cheng, Joseph R. Wang, Hong-Jian Wei, Win-Ping Deng

**Affiliations:** 1 Graduate Institute of Biomedical Materials and Engineering, College of Biomedical Engineering, Taipei Medical University, Taipei 110, Taiwan; d225101001@tmu.edu.tw; 2 Stem Cell Research Center, College of Oral Medicine, Taipei Medical University, Taipei 110, Taiwan; 3 School of Dentistry, College of Oral Medicine, Taipei Medical University, Taipei 110, Taiwan; pemg@tmu.edu.tw (B.-Y.P.); g4808@tmu.edu.tw (H.-C.C.); 4 Department of Dentistry, Taipei Medical University Hospital, Taipei 110, Taiwan; 5 Division of Gastroenterology and Hepatology, Department of Internal Medicine, Taipei Medical University Hospital, Taipei 110i, Taiwan; chunchao@tmu.edu.tw; 6 Division of Gastroenterology and Hepatology, Department of Internal Medicine, Taipei Medical University School of Medicine, Taipei 110, Taiwan; 7 Ceramics and Biomaterials Research Group, Advanced Institute of Materials Science, Ton Duc Thang University, Ho Chi Minh City 700000, Vietnam; navneet.kumar.dubey@tdtu.edu.vn; 8 Faculty of Applied Sciences, Ton Duc Thang University, Ho Chi Minh City 700000, Vietnam; 9 Department of Neurosurgery, Taipei Medical University Hospital, Taipei 110, Taiwan; d102092012@tmu.edu.tw; 10 Division of Neurosurgery, Department of Surgery, School of Medicine, College of Medicine, Taipei Medical University, Taipei 11031, Taiwan; 11 Department of Periodontics, College of Dental Medicine, Columbia University, New York 10032, USA; jrw2166@cumc.columbia.edu; 12 School of Dental Technology, College of Oral Medicine, Taipei Medical University, Taipei 110, Taiwan; 13 Graduate Institute of Basic Medicine, Fu Jen Catholic University, New Taipei City 110, Taiwan

**Keywords:** adipose-derived stem cells, protein vaccine, T cell responses, tumor microenvironment, antigen processing

## Abstract

Cancer is a leading cause of mortality and a major public health problem worldwide. For biological therapy against cancer, we previously developed a unique immunotherapeutic platform by combining mesenchymal stem cells with an antigen-specific protein vaccine. However, this system possesses a few limitations, such as improperly immortalized mesenchymal stem cells (MSCs) along with transfected oncogenic antigens in them. To overcome the limitations of this platform for future clinical application, we freshly prepared primary adipose-derived stem cells (ADSCs) and modified the E7’ antigen (E7’) as a non-oncogenic protein. Either subcutaneously co-inoculated with cancer cells or systemically administered after tumor growth, ADSC labeled with enhanced green fluorescent protein (eGFP) and combined with modified E7’ (ADSC-E7’-eGFP) cells showed significant antitumor activity when combined with the protein vaccine in both colon and lung cancer in mice. Specifically, this combined therapy inhibited tumor through inducing cell apoptosis. The significantly reduced endothelial cell markers, CD31 and vascular endothelial growth factor (VEGF), indicated strongly inhibited tumor angiogenesis. The activated immune system was demonstrated through the response of CD4+ T and natural killer (NK) cells, and a notable antitumor activity might be contributed by CD8+ T cells. Conclusively, these evidences imply that this promising immunotherapeutic platform might be a potential candidate for the future clinical application against cancer.

## 1. Introduction

Despite tremendous advances in both clinical and basic research, cancer remains one of the leading causes of mortality and a major public health problem worldwide [1]. Many tumors continue to have a poor prognosis even after conventional therapies such as surgical treatment, radiation, and chemotherapy. Thus, biological therapy has become a novel and promising strategy for cancer treatment. The definition of biological therapy is the use of living organisms, substances derived from living organisms, or laboratory-produced versions of such substances to treat disease [2]. Current biological therapy for cancer includes gene therapy, targeted therapy, and immunotherapy such as vaccines, cytokines, and antibodies. Nonetheless, one of the major obstacles encountered in cancer biological therapy is the inefficient delivery of therapeutic agents into the tumor lesions, especially the small metastatic or relapsed tumors. Previously, we have demonstrated that, after systemic delivery, bone marrow-derived mesenchymal stem cells (BMSCs) could migrate to microscopic tumor lesions and engraft into tumor stroma [3]. The tumor tropism of mesenchymal stem cells (MSCs) has been established in various cancer types [4,5,6,7,8], which, thus, made MSCs potential vehicles for delivering anti-cancer agents [9,10,11,12].

Taking advantage of the tumor-homing and infiltrating abilities of MSCs, we developed a unique cancer immunotherapeutic platform by combining MSCs with an antigen-specific protein vaccine. We utilized human papillomavirus type 16 (HPV-16) E6/E7-immortalized BMSCs as an E7 antigen-delivering vehicle to mediate the antitumor effect of the E7 antigen-specific protein vaccine on non-E7 expressing fibrosarcoma cells [13]. However, there are some limitations of the previous platform for future clinical application. First, the MSCs used in the previous platform were immortalized by HPV-16 E6/E7 genes, which raises safety concerns in clinical utilization. Although the immortalized MSCs have been characterized to be non-tumorigenic in advance [14], such immortalized MSCs are inappropriate for clinical application due to the unforeseen outcome of long-term usage. Hence, in the current study, we aim to establish the antigen-delivering vehicle with a modified-E7 antigen, which is a non-oncogenic protein, and freshly prepared primary MSCs. Moreover, we used adipose tissue as the cell source of MSCs in this study. The main advantage of adipose-derived stem cells (ADSCs), over MSCs derived from other sources (e.g., from bone marrow), is that they can be easily and repeatable harvested by utilizing minimally invasive techniques with low morbidity [15].

Second, by using MSCs, we successfully expanded the therapeutic spectrum of the E7-specific protein vaccine in non-E7 expressing tumors in the previous study [13]. To prove that the combination of antigen-delivering MSCs with an antigen-specific protein vaccine has the potential to serve as a universal treatment for different cancer types, we aim to expand the therapeutic spectrum of this unique cancer immunotherapeutic platform in colon cancer and lung cancer, which are two of the most common cancers worldwide [16]. Furthermore, the immunological mechanisms underlying tumor inhibition are unclear. Hence, in the current study, we also aim to investigate the role of different immune cells involved in the anti-tumor responses.

## 2. Results

### 2.1. Modified-E7 (E7’) Transduction of Primary ADSCs with a Lentiviral Vector

**Figure 1:**
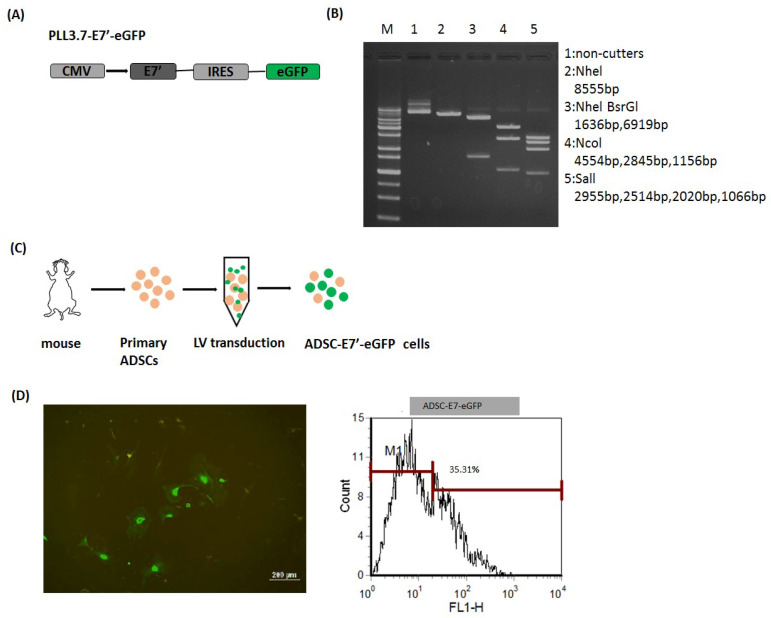
Establishment of ADSC labeled with enhanced green fluorescent protein (eGFP) and combined with modified E7’ (ADSC-E7’-eGFP). (**A**) Schematic diagram of pLL3.7-E7’-eGFP construction; (**B**) agarose gel electrophoresis of plasmid pLL3.7-E7’-eGFP (M: 1 kb DNA ladder; lane 1: Undigested plasmid; lane 2: uNheI (8555 bp); lane 3: NheI and BsrGI (1636 bp and 6919 bp); lane 4: bNcoI (4554 bp, 2845 bp, and 1156 bp); lane 5: bSalI (2955 bp, 2514 bp, 2020 bp, and 1066 bp)); (**C**) illustration of lentiviral transduction of primary ADSCs; and (**D**) fluorescence microscopy and flow cytometric analysis of ADSC-E7’-eGFP cells.

Previously, we have utilized E6/E7-immortalized MSCs as an E7 antigen-delivering vehicle to successfully expand the therapeutic spectrum of the E7 antigen-based protein vaccine (PE(ΔIII)-E7-KDEL3) on non-E7 expressing tumors [13]. However, the long-term usage of such immortalized MSC may cause several unforeseen outcomes, such as tumorigenicity, outcompeting with endogenous MSCs, and different immunomodulatory effects, raising the safety concerns in clinical utilization. Hence, in order to mimic future clinical application, we then conducted this cancer immunotherapeutic platform with freshly prepared primary stem cell and syngeneic tumor models. Moreover, to exclude the oncogenic activity of HPV-16-E7, E7 was modified to a non-oncogenic protein (indicated as E7’) with mutations in the Rb binding site. First, we constructed the lentiviral vector, pLL3.7-E7’-eGFP, contained CMV-promoter-driven E7’, and enhanced green fluorescent protein (eGFP) genes that were separated by the internal ribosomal entry site (IRES) (Figure 1A). The constructed pLL3.7-E7’-eGFP plasmid was verified by restriction enzyme digestion and gel electrophoresis (Figure 1B). The E7 antigen-delivering vehicle, ADSC-E7’-eGFP, was then established by lentiviral transduction, as illustrated in Figure 1C. Primary ADSCs were isolated from the abdominal fat of mice and then transduced with the E7’-eGFP gene by the pLL3.7-E7’-eGFP lentiviral vector. The expression of GFP in the ADSC-E7’-eGFP cell was validated by fluorescent microscopic and flow cytometry (Figure 1D). This readymade ADSC-E7’-eGFP was then used for further experimental studies.

### 2.2. ADSC-E7’-eGFP–PE(ΔIII)-E7-KDEL3 Combined Treatment Inhibits the Tumor Growth of Colon and Lung Cancer Cells in Mice

**Figure 2:**
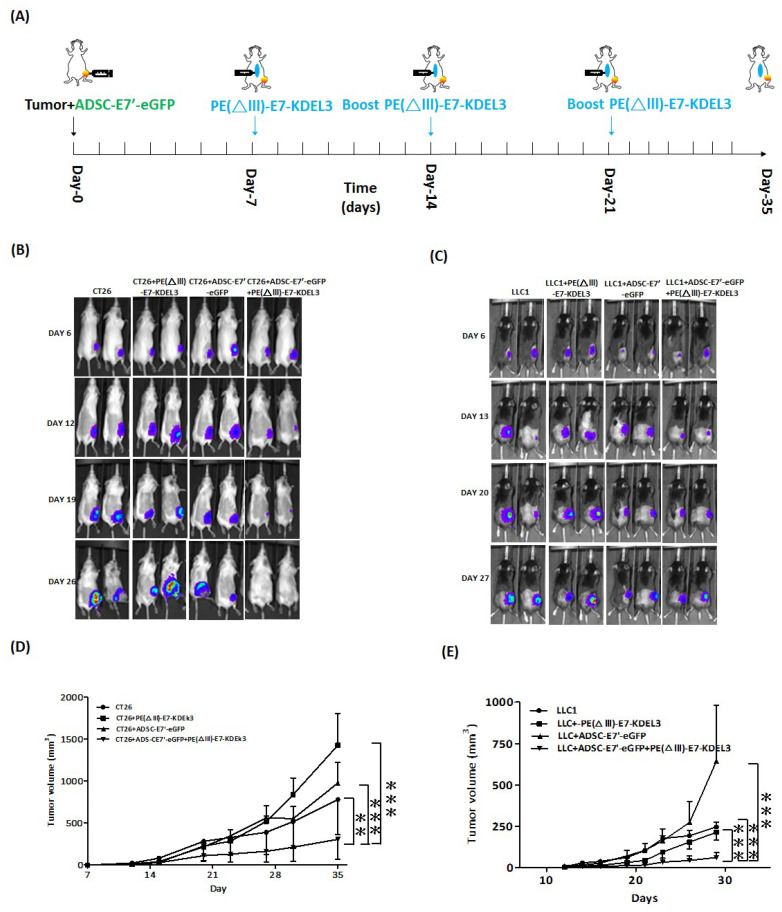
The tumor inhibition of the combined treatment by subcutaneously inoculated ADSC-E7’-eGFP and the protein vaccine. (**A**) Time course of the experiment. Representative bioluminescence images of mice subcutaneously injected with (**B**) 2 × 10^5^ CT26 cells with indicated treatment or (**C**) 2 × 10^5^ LLC1 cells with indicated treatment. Tumor volume measurements of syngeneic tumor models were conducted at indicated days after subcutaneous injection of (**D**) CT26 cells or (**E**) LLC1 cells; ** *p* < 0.01; *** *p* < 0.001 using two-way ANOVA.

To examine the inhibiting effects of the combined treatment of ADSC-E7’-eGFP and PE(ΔIII)-E7-KDEL3 on tumor growth, we first subcutaneously co-inoculated ADSC-E7’-eGFP cells with cancer cell lines in syngeneic mice. Before the experiments, the primary ADSCs were harvested and transduced to ADSC-E7’-eGFP cells (Figure 1C). Seven days after cell injection, the mice were administered with first immunization of PE(ΔIII)-E7-KDEL3, and then followed by two booster shots at day 14 and 21 (Figure 2A). Two cancer cell lines, colon tumor 26 (CT26) cell and Lewis lung carcinoma (LLC1) cell, were used to evaluate the immunotherapeutic spectrum of the combined treatment. Both cells lines were transduced with firefly luciferase, and non-invasive bioluminescence imaging was conducted to monitor the extent and relative growth of the tumors in mice. Animals receiving the combined treatment revealed a gradual decrease in signal intensity, reflecting reduced tumor burden over time (Figure 2B,C). The quantitative results showed that the tumor volumes of CT26 and LLC1 cells in the combined treatment group were significantly smaller than those in other groups (Figure 2D,E).

### 2.3. Systemic Administration of ADSC-E7’-eGFP in Combination with PE(ΔIII)-E7-KDEL3 Reduces the Growth of Colon and Lung Cancer Cell Induced Tumors

**Figure 3:**
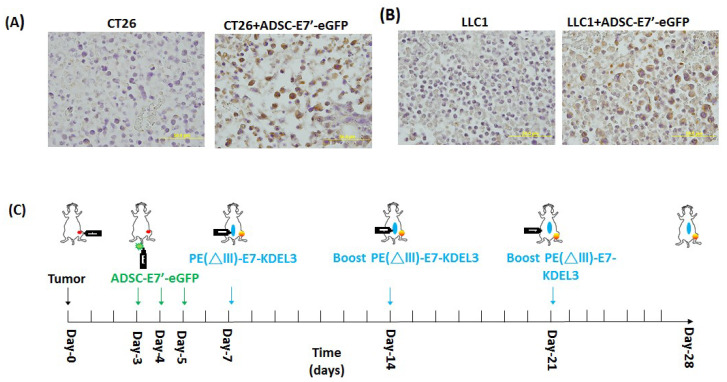

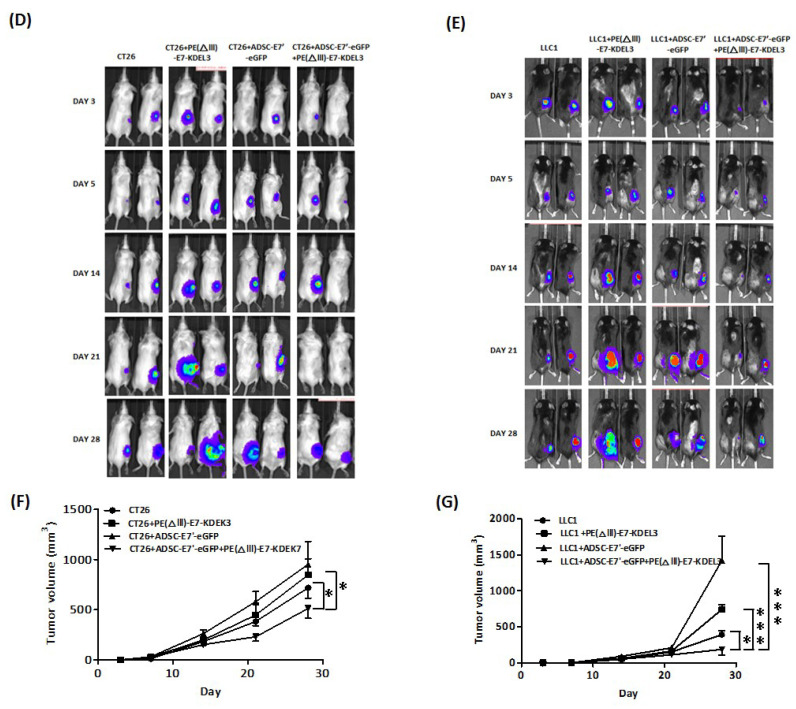
The tumor inhibition of the combined treatment by the systemic administration of ADSC-E7’-eGFP and the protein vaccine. The GFP immunohistochemical staining of (**A**) the CT26 tumor with or without the systemic administration of ADSC-E7’-eGFP cells; or (**B**) the LLC1 tumor with or without the systemic administration of ADSC-E7’-eGFP cells. (**C**) Time course of the experiment. Two representative bioluminescence images of mice subcutaneously injected with (**D**) 2 × 10^5^ CT26 cells with indicated treatment; or (**E**) 2 × 10^5^ LLC1 cells with indicated treatment. Tumor volume measurements of syngeneic tumor models were conducted at indicated days after subcutaneous injection of (**F**) CT26 cells; or (**G**) LLC1 cells; * *p* < 0.05, ** *p* < 0.01; and *** *p* < 0.001 using two-way ANOVA.

Data from both the bioluminescence images and the tumor volume quantitation suggested that the ADSC-E7’-eGFP–PE(ΔIII)-E7-KDEL3 combined treatment posed a strong inhibitory effect on the tumor growth of colon and lung cancer cells (Figure 2). We then evaluated the therapeutic effect of systemically administered ADSC-E7’-eGFP to mimic future clinical application. In the beginning the primary ADSC were harvested and freshly prepared as ADSC-E7’-eGFP cells (Figure 1C). Subsequently, we validated, by staining the tumor sections, that the intravenously injected ADSC-E7’-eGFP cells indeed homed to the tumor stroma. The results showed that the tumor section that had intravenous injection of ADSC-E7’-eGFP cells highly presented the signals of GFP staining, whereas no GFP signal was observed in CT26 and LLC1 groups (Figure 3A,B). We first injected tumor cells subcutaneously in mice (day 0). Three days after tumor inoculation, tumor-bearing mice received daily injections of ADSC-E7’-eGFP, from day 3 to day 5, via intravenous injection. Mice were then immunized by PE(ΔIII)-E7-KDEL3 on day 7, and followed by two booster shots at day 14 and 21 (Figure 3C). Bioluminescence images were obtained on day 3, 5, 14, 21, and 28 post tumor inoculation (Figure 3D,E). Animals that received the combined treatment showed a gradual decrease in imaging signal intensity, reflecting reduced tumor burden over time. The in vivo imaging results were then evidenced by the volume of the subcutaneous tumor. Quantitative result showed that, in both CT26 and LLC1 cells, tumor volumes in the combined treatment group were significantly smaller than those in other groups (Figure 3F,G). Data from both the bioluminescence image and the tumor volumes quantitation suggested that the ADSC-E7’-eGFP–PE(ΔIII)-E7-KDEL3 combined treatment posed a strongly inhibitory effect on the tumor growth of colon and lung cancer cells, not only in subcutaneous but also in systemic administration

### 2.4. Systemic Administration of ADSC-E7’-eGFP in Combination with PE(ΔIII)-E7-KDEL3 Enhances Apoptosis in Tumors

**Figure 4:**
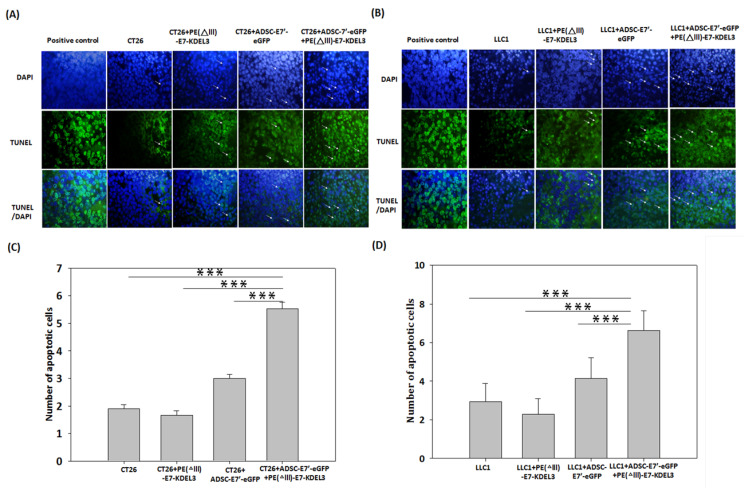
Evaluation of apoptosis in tumor tissues by TUNEL staining. Representative fluorescence images of (**A**) the CT26 tumor with different treatments; or (**B**) the LLC1 tumor with different treatments. Apoptotic-positive cells were shown in green (arrows) and the cellular nucleus was stained by DAPI and shown in blue. (**C**,**D**) The number of apoptotic-positive cells in the microscopic fields were calculated. The quantitative results are presented as means + standard error of means (SEM); *** indicates *p* < 0.001 using unpaired *t*-test with Welch’s correction. Magnification A,B: 50 µm.

Tumor inhibition is caused by several events, including cell cycle arrest, cell apoptosis, anti-angiogenesis, and immunosurveillance. To determine whether apoptosis is involved in the inhibitory effect, on tumors, of the systemically administered combined treatment of ADSC-E7’-eGFP with PE(ΔIII)-E7-KDEL3, a TUNEL assay was conducted after 28 days of CT26 and LLC1 inoculation. Representative fluorescence images show that the ADSC-E7’-eGFP–PE(ΔIII)-E7-KDEL3 combined treatment group presented more apoptotic cells (green) than other groups, in both CT26 and LLC1 tumors (Figure 4A,B). Quantitative results demonstrated that, in both cancer types, the number of apoptotic cells was significantly higher in ADSC-E7’-eGFP–PE(ΔIII)-E7-KDEL3 combined treatment than that in other groups (Figure 4C,D). The ADSC-E7’-eGFP–PE(ΔIII)-E7-KDEL3 combined treatment showed a 2- to 3-fold increase in apoptosis compared to control group, which imply that apoptosis induced by the combined treatment may contribute to tumor inhibition.

### 2.5. Inhibition of Tumor Angiogenesis by the ADSC-E7’-eGFP–PE (ΔIII)-E7-KDEL3 Combined Treatment

**Figure 5:**
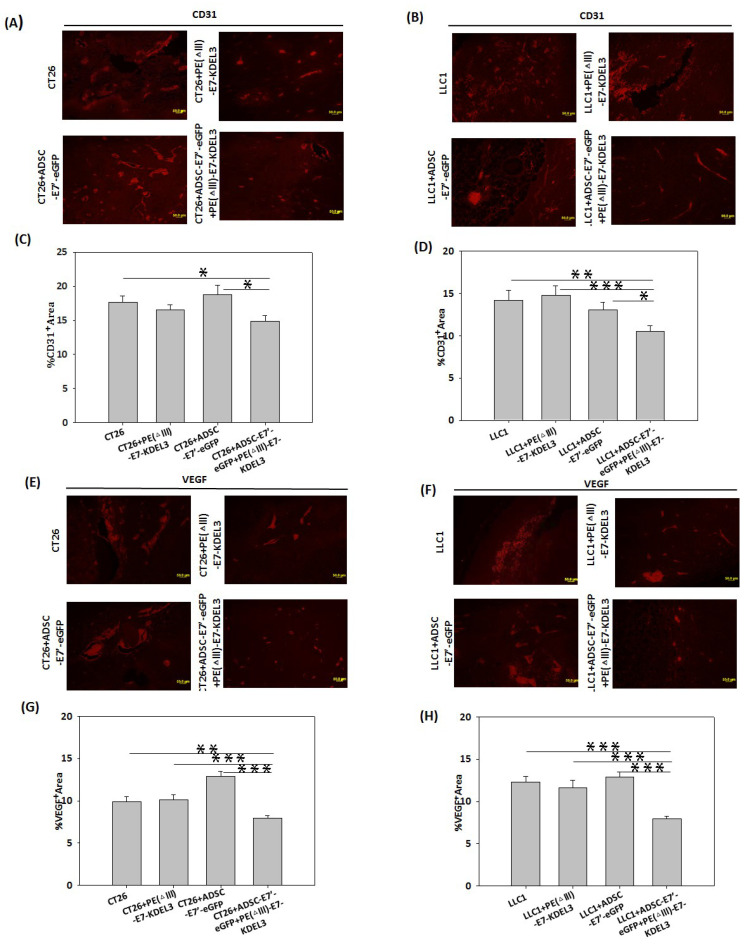
Histological assessment of tumor angiogenesis. Representative fluorescence images of tumor sections from mice inoculated with (**A**) CT26 cells or (**B**) LLC1 cells, which were stained with anti-CD31 (red) after 28 days to detect tumor-associated blood vessels. The quantitative results were determined by the average blood vessel area per microscopic field in tumor sections from mice inoculated with (**C**) CT26 cells or (**D**) LLC1 cells. Values are means + SEM; * *p* < 0.05; ** *p* < 0.01, *** *p* < 0.001 using unpaired *t*-tests (*n* = 3). Representative fluorescence images of tumor sections from mice inoculated with (**E**) CT26 cells or (**F**) LLC1 cells were stained with anti-VEGF (vascular endothelial growth factor) (red) to detect tumor vascularization. The quantitative results were determined by the average VEGF expression area per microscopic field in tumor sections from mice inoculated with (**G**) CT26 cells or (**H**) LLC1 cells. Values are means + SEM; ** *p* < 0.01, *** *p* < 0.001 using unpaired *t*-tests (*n* = 3). Magnification A,B,E,F: 50 µm.

To determine whether angiogenesis is also involved in the antitumor effect of the systemically administered combined treatment of ADSC-E7’-eGFP with PE(ΔIII)-E7-KDEL3, the endothelial cell marker CD31 and vascular endothelial cell growth factor (VEGF) were detected 28 days after tumor inoculation. The tumor sections were stained and observed under fluorescent microscope (Figure 5A,B and Figure 5E,F). The results quantitatively revealed that both the CD31 and VEGF expression of the ADSC-E7’-eGFP–PE(ΔIII)-E7-KDEL3 combined treatment groups were significantly lower than the other single treatment or control groups (Figure 5C,D and Figure 5G,H), suggesting that the tumor regression induced by the combined treatment might be due to the inhibition of angiogenesis.

### 2.6. ADSC-E7’-eGFP–PE(ΔIII)-E7-KDEL3 Combined Treatment Contributes to Tumor Regression by Immune System Activation

**Figure 6:**
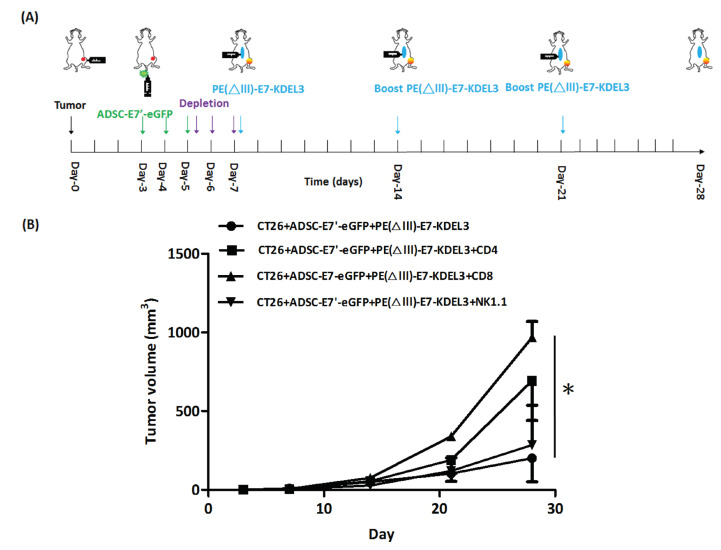
In vivo antibody depletion reduces the antitumor effect of the ADSC-E7’-eGFP–PE(ΔIII)-E7-KDEL3 combined treatment. (**A**) Time course of the experiment; (**B**) tumor volume measurements of syngeneic tumor models with different treatments were conducted at indicated days after the subcutaneous injection of CT26 cells; * *p* < 0.05 using two-way ANOVA.

The above-mentioned combined treatment of ADSC-E7’-eGFP and the PE(ΔIII)-E7-KDEL3 protein vaccine showed strong inhibition of tumors. The PE(ΔIII)-E7-KDEL3 protein vaccine has been demonstrated to stimulate the activation of specific CD4+ and CD8+ T cells [17]. In this study; however, we specifically conducted a syngeneic animal model to examine the real immune environment in which the CD4, CD8, and natural killer (NK) cells play important roles in ADSC-E7’-eGFP-mediated tumor inhibition. Therefore, the antibody depletion of immune cells was used in the combined treatment mouse model. The experiment was divided into four groups, the ADSC-E7’-eGFP–PE(ΔIII)-E7-KDEL3 group (control group), the ADSC-E7’-eGFP–PE(ΔIII)-E7-KDEL3 with CD4 depletion group, the ADSC-E7’-eGFP–PE(ΔIII)-E7-KDEL3 with CD8 depletion group, and the ADSC-E7’-eGFP–PEΔ (III)-E7-KDEL3 with NK depletion group. First, mice were subcutaneously inoculated with CT26 cells at day 0, and then ADSC-E7’-eGFP cells were intravenously injected thrice on days 3 to 5. We then conducted antibody depletion of CD4+, CD8+, or NK cells, 3 times, on days 5 to 7. The first injection of PE(ΔIII)-E7-KDEL3 was done on day 7, and followed by booster shots on day 14 and 21 (Figure 6A). The tumor volume results showed that the tumor inhibition effect of the control group was significant on day 28, which was then diminished in each immune cell depletion group, especially on the depletion of CD8+ T cell group (Figure 6B). These results suggested that the CD4+T, CD8+T, and NK cells are all essential for the antitumor immunity of the ADSC-E7’-eGFP–PE(ΔIII)-E7-KDEL3 combined treatment, and that the effect of tumor inhibition might be achieved by the CD8+ T cells.

## 3. Discussion

Mesenchymal stem cell (MSC)-based therapies offer a promising strategy that provides alternative therapeutic solutions, for various diseases, to repair and regenerate tissues and organs [18]. In addition, MSCs are emerging as potential vehicles for delivering anti-cancer agents due to their inherent tumor homing capacity [3]. The most common strategy of MSC-based therapies for cancer is to genetically modify them with tumor suppressor genes, anti-angiogenic agents, and immunomodulating cytokines [19]. Other therapeutic approaches include MSC-mediated gene directed enzyme prodrugs, or their loading with anticancer drugs/ nanoparticles [20]. Previously, we have developed a unique cancer immunotherapeutic platform by combining tumor-targeting MSCs with a protein vaccine [13]. We utilized E6/E7-immortalized MSCs as an E7 antigen-delivering vehicle to expand the therapeutic spectrum of the E7 antigen-based protein vaccine. However, such immortalized MSCs are associated with many safety concerns and are inappropriate for clinical application. Therefore, in this study, we re-established the cancer immunotherapeutic platform by using freshly prepared primary ADSCs and a syngeneic tumor model to mimic the future clinical application. Additionally, we modified the E7 antigen of ADSCs (indicated as E7’), as a non-oncogenic protein with mutation, in the R6 binding site. Either subcutaneously co-inoculated with cancer cells or systemically administered after tumor growth, the ADSC-E7’-eGFP showed significant antitumor activity when combined with the protein vaccine.

MSCs are multipotent stem cells that can self-renew and differentiate into a variety of cell lineages, and were first isolated and identified from bone marrow [21]. Currently, MSCs can routinely be isolated from several tissue niches in the body, such as fat, muscle, tendon, umbilical cord blood and amniotic fluid [22]. MSCs from distinct origins have revealed variable growth potentials; however, they share a similar surface marker profile and multilineage differentiation capacity. It is well documented that adipose tissue is a rich source of MSCs, and, compared to other sources, the clinical procedure to harvest them is minimally invasive and less painful [23]. Hence, due to their convenient acquisition and innate tumor tropism, ADSCs have attracted much recent attention as a promising vehicle for delivering anti-cancer molecules. Studies have shown that the genetic modification of ADSCs with interferon β (IFN-β) inhibits the growth of melanoma cells in vitro as well as in vivo. Moreover, the antitumor activity of IFN-β-expressed ADSCs was increased when combined with low-dose cisplatin [24]. The antitumor effect of ADSCs modified with tumor necrosis factor-related apoptosis-inducing ligand (TRAIL), one of the most promising therapeutic pro-apoptotic cytokines, has also been demonstrated in several types of tumors [25,26,27,28]. In addition to IFN-β or TRAIL, there are several other tumor-suppressor genes and cytokines with anticancer activities, such as CXCL10 [29], IL-12 [30], and pigment epithelium-derived factor (PEDF) [31], which are utilized for the genetic modification of ADSCs. These ADSCs have also been employed as delivery vehicles in prodrug cancer gene therapy, including herpes simplex virus type 1 thymidine kinase (HSV1-TK)/ganciclovir (GCV) [32], cytosine deaminase (CD)/5-fluorocytosine (5-FC) [33], cytosine deaminase-uracil phosphoribosyl transferase (CD/UPRT)/5-FC [34], and rabbit carboxylesterase(rCE)/irinotecan-7-ethyl-10-[4-(1-piperidino)-1-piperidino]carbonyloxycamptothecin (CPT-11) [35]. Based on previous studies, demonstrating the therapeutic potential of modified MSCs, we isolated primary ADSCs and transfected them with the E7’ vector in order to utilize it as an antigen-delivering vehicle in our cancer immunotherapeutic platform.

An ideal cancer treatment should be capable to specifically discriminate normal and cancer cells and systemically eradicate multiple tumor lesions in the body. Hence, due to the induction of tumor-specific reactions, antigen-specific immunotherapy is a potentially attractive cancer treatment modality. However, such a strategy was limited by tumor variants that lacked the target antigen and resisted the specific immune response. To overcome the main obstacles encountered in cancer immunosurveillance, we previously utilized immortalized BMSCs as an E7 antigen-delivering vehicle to mediate the antitumor effect of the E7 antigen-based protein vaccine on non-E7 expressing fibrosarcoma cells [13]. In this study, we further expanded the therapeutic spectrum of the E7 antigen-based protein vaccine in colon and lung cancer cells by using E7’-expressing ADSCs. In a study by Liao et al., the PE(ΔIII)-E7-KDEL3 protein vaccine was proved to elicit its antitumor effect through all venues of immunological responses, including CD4+ T, CD8+ T, and natural killer (NK) cells [17]. We also previously showed that the combined treatment of MSCs and PE(ΔIII)-E7-KDEL3 induced CD4+ T cell activation via major histocompatibility complex (MHC) class II molecules in vitro [13]. Nonetheless, here we showed that CD4+ T, CD8+ T, and NK cells contributed to the antitumor activity of the combined treatment of ADSCs and the protein vaccine by in vivo antibody depletion. We also suggest that the antitumor effect of the combined treatment might be achieved by CD8+ T cells. Although CD4+ T cells and MHC class II molecules are associated with the tumor antigen-specific immune response [36,37,38,39], the tumor antigens have been shown to present predominantly in association with MHC class I molecules, and to be recognized by tumor-specific CD8+ T cells [40,41]. In addition, MSCs have been demonstrated to exert antigen-presenting properties to activate CD4+ T cells via MHC class II molecules upon interferon gamma (IFN-γ) stimulation [42,43]. François et al. further identified that MSCs could cross-present exogenous antigens and induce an effective CD8+ T-cell immune response both in vitro and in vivo [44]. These data strongly suggest that MSCs could behave as conditional antigen-presenting cells (APCs) to activate antigen-specific immune responses. This unique property of MSCs render them as a potential cell-based immune biopharmaceutic for cancer treatment.

Taken together, we demonstrated that the freshly prepared primary ADSCs could be genetically modified as an antigen-delivering vehicle to expand the therapeutic spectrum of the antigen-specific protein vaccine. Further, the combination of ADSC-E7’-eGFP cells with the PE(ΔIII)-E7-KDEL3 protein vaccine significantly inhibited the tumor growth of CT26 and LLC1 cells, through apoptotic activity in addition to the reduction of tumor angiogenesis. The activated immune system was mediated by CD4+ T and NK cells, whereas the antitumor activity was mainly contributed to by CD8+ T cells. These results provide strong evidences that support this promising immunotherapeutic platform for future clinical application in cancer therapy.

## 4. Materials and Methods

### 4.1. Cell Lines

LLC1 (ATCC CRL-1642, ATCC, Taipei, Taiwan) Lewis lung carcinoma cells and CT26 (ATCC CRL-2638, Taipei, Taiwan) colon cancer cells were infected with FUW-Luc-mCh-puro lentiviral particles and cultured in RPMI 1640 medium supplemented with 10% fetal bovine serum (FBS, Gibco, Mexico City, Mexico), 100 units/mL penicillin, 100 μg/mL streptomycin, 0.25 μg/mL amphotericin B, and 2 μg/mL puromycin in a humidified atmosphere, with 5% CO_2_ at 37 °C, to stably express firefly luciferase and mCherry fluorescent protein.

### 4.2. Isolation, Culture, and Lentiviral Transduction of ADSCs

ADSCs were isolated and cultured as previously described [45]. The E7’ antigen and enhanced green fluorescent protein were expressed in ADSCs by using lentiviral transduction. The lentiviral vector (pLL3.7-E7’-eGFP) and the packaging constructs (pMDL g/p RRE, p RSV-REV, and pMD2.G) were transfected into 293FT cells (Invitrogen, Fisher Scientific, Carlsbad, CA, USA) with Lipofectamine 2000 Transfection Reagent (Invitrogen). Infectious viral particles were collected 48 h after transfection. ADSCs were infected with appropriate virus titers in media containing 8 μg/mL polybrene. After 24 h of infection, the medium of ADSCs were replaced with complete growth medium.

### 4.3. Preparation and Vaccination of Protein Vaccines

The preparation of the PE(ΔIII)-E7-KDEL3 protein vaccine and the vaccination in mice were conducted as previously described [13]. Briefly, mice were immunized with 0.1 mg/mouse PE(ΔIII)-E7-KDEL3, mixed with 10% ISA206 adjuvant, by subcutaneous injection into the backs of the mice. These animals were then boosted, subcutaneously, 1 and 2 weeks later using the same regimen.

### 4.4. Animal Studies

All animal studies were approved by The Institutional Animal Care and Use Committee (IUCAC) of Taipei Medical University (Approval no. LAC-2014-004; 16 January 2014). Six-week-old female BALB/c and female C57BL/6 mice were purchased from the National Laboratory Animal Center and the National Applied Research Laboratories (Taipei, Taiwan). The mice were housed under pathogen-free conditions and fed autoclaved food and water. Tumor xenografts were established by subcutaneous injection of 2 × 10^5^ LLC1 cells or 2 × 10^5^ CT26 cells. ADSCs were administered by subcutaneous co-inoculation with cancer cells at the same time, or by intravenous injection 3 days later. Mice were first immunized with the PE(ΔIII)-E7-KDEL3 protein vaccine 7 days after tumor inoculation, and received boost shots 1 and 2 weeks later.

### 4.5. Bioluminescence Imaging (BLI)

BLI of animals was performed with an IVIS Imaging System 200 Series (PerkinElmer, Waltham, MA, USA) and quantitated with Living Image® software by measuring photon flux (photons/s/cm2/steradian) in regions of interest drawn around appropriate signals. For in vivo BLI, anesthetized mice (*n* = 5) were injected intraperitoneally with 75 mg/kg of D-Luciferin, and images were acquired 2 to 5 min after injection. Acquisition times were 2 min initially, and were reduced in accordance with signal intensity to avoid saturation.

### 4.6. Histology

Tumor tissues were fixed in 10% neutral buffered formalin, processed, and embedded in paraffin. For immunohistochemistry, tissue sections were incubated overnight with an anti-GFP antibody. The primary antibodies were detected using ABC and DAB substrate kits (Vector, Burlingame, CA, USA) and the sections were counterstained with hematoxylin. For immunofluorescence, issue sections were incubated overnight with anti-CD31 or anti-VEGF PE-conjugated antibodies.

### 4.7. TUNEL Assay

After 28 days of CT26 and LLC1 inoculation, a TUNEL assay was performed using DeadEnd™ Fluorometric TUNEL System (Promega, Madison, WI, USA) according to the manufacturer’s instructions. Briefly, subcutaneous tumor sections from the mice of the combined-treatment group or the stem cells only group were made permeable with 20 μg/mL of proteinase K for 10 minutes at room temperature, and the fragmented DNA was labeled using the TdT (terminal deoxynucleotidyl transferase) reaction mixture, containing fluorescein-12-dUTP, for 1 hour at 37 °C, according to supplier recommendations. The slides were mounted in VECTASHIELD Antifade Mounting Medium with DAPI (Vector). The results were expressed quantitatively by the number of apoptotic cells per field of view.

### 4.8. In Vivo Antibody Depletion

For in vivo antibody depletion, mice were treated via intraperitoneal injection with 500 μg/day of anti-CD4 (clone GK1.5), anti-CD8 (clone 53-6.7), or anti-NK1.1 (clone PK136) antibodies for 3 consecutive days.

### 4.9. Statistical Analysis and Replicates

The sizes of the sample groups in all data were at least *n* = 5, unless otherwise indicated. All data presented were representative of at least three independent experiments that yielded similar results. Statistical analyses were performed using GraphPad Prism 5 (https://www.graphpad.com/support/prism-5-updates/).

## 5. Conclusions

This study demonstrated that a combined therapy of ADSC-E7’-eGFP–PE(ΔIII)-E7-KDEL3 activated the immune system through the response of CD4+ T and NK cells. Additionally, a notable antitumor activity might be contributed by CD8+ T cells. These evidences imply that this promising immunotherapeutic platform might be a potential candidate for the future clinical application against cancer.
